# Near-superhydrophobic silicone microcapsule arrays encapsulating ionic liquid electrolytes for micro-power storage assuming use in seawater

**DOI:** 10.1038/s41598-022-22891-w

**Published:** 2022-10-29

**Authors:** Kaede Iwasaki, Tsuyoshi Yoshida, Masayuki Okoshi

**Affiliations:** Department of Electrical and Electronic Engineering, National Defense Academy, 1-10-20 Hashirimizu, Yokosuka, Kanagawa 239-8686 Japan

**Keywords:** Energy science and technology, Engineering, Materials science

## Abstract

Micro-energy storage, which is convenient for combination with energy harvesting, is known to be realized by microencapsulation with various shell materials, its application is limited to land. Here, we succeeded in fabricating a silicone microcapsule array encapsulating an ionic liquid electrolyte that can store minute power in NaCl solution as well as a minute power generation method. The ArF excimer laser-irradiated silicone rubber underneath silica microspheres was photochemically and periodically swelled by the photodissociation of silicone. Accompanied by the microswellings, the lower molecular weight silicones generated were ejected along a curvature of each the microsphere to enclose the microspheres. After the chemical etching, the silicone microcapsule arrays became hollow. Moreover, each the hollow silicone microcapsule could entrap an ionic liquid in a vacuum. In addition, the silicone microcapsules before and after the encapsulating ionic liquid showed a superhydrophobic or near-superhydrophobic property. As a result, the silicone microcapsule arrays could be confined in a uniform air gap of electrically insulated region in NaCl solution. This means that each the silicone microcapsule encapsulating ionic liquid as electrolytes enables to function as an electric double layer capacitor for micro-power storage, aiming to connect with Internet of Things devices that work under seawater.

## Introduction

Microencapsulation has a long history, starting with the creation of living cells. Most unicellular plants or animals are living examples of microencapsulation^1^. The most important functions of microencapsulation are the protection of internal substances and the control of the flow of substances across the cell membrane. On the other hand, a carbonless copy paper was an early example of successful artificial application of microencapsulation^2^. Currently, the microencapsulation can be defined as a process in which small particles or droplets of the active agent are surrounded by a coating or embedded in a polymeric material, to give small capsules that may range from submicrons to several millimeters with many useful properties^3^. The enclosed material represents the core, and the material that covers around the core is called the shell or shell wall.

Microencapsulation is also an important technology from the perspective of materials micro/nano-processing and has progressed to expand its applications in recent years^4,5^. The reasons for requiring the microencapsulation are not the same, but basically it is needed to isolate a core material from its surrounding, in addition to the releasing it when and where it is needed. One of the applications that makes good use of its characteristics is a drug delivery system^6^. The drug delivery system is to control the distribution of drugs in the body quantitatively, spatially, and temporally. Numerous different microencapsulation for drug delivery systems have been reported^7–9^. As another effective application, the technology is used for self-healing to develop unique coating methods^10–12^. In any cases, the shell wall must be ruptured at the time of use. On the other hand, microencapsulation can be also used for storage of materials as micro/nanocontainers^13,14^. In astronomy, in the asteroid Ryugu’s returned sample, it was found that an aliphatic carbon-rich organic matter was concentrated in coarse-grained hydrous silicate minerals. This means that the coarse-grained hydrous silicate minerals as a shell wall became cradles of organic matter and water and were transported to earth intact^15^. For energy application, phase change materials have been enclosed in various shell walls for thermal energy storage^16–20^. In this case, however, the fabricated microcapsules are basically separated and independent. In addition, in some reports, it seems to be difficult to control the shape and size uniformly.

In this paper, a uniform shape and size of hollow spherical silicone microcapsule arrays is successfully fabricated on a silicone rubber by the 193 nm ArF excimer laser-induced photodissociation of silicone rubber. Moreover, each the fabricated hollow silicone microcapsule can entrap an ionic liquid in a vacuum. As the fabricated silicone microcapsule arrays before and after the encapsulating ionic liquid are fixed to the silicone rubber, the sample surfaces show a superhydrophobic or near-superhydrophobic property. As a result, the silicone microcapsule arrays encapsulating the ionic liquid as electrolytes can be confined in a uniform air gap of electrically insulated region in sodium chloride (NaCl) aqueous solution, for micro-power storage application. In fact, based on the difference in electrochemical potentials, a combination of our previous method^21^ allows electric voltages to be generated simultaneously in the air gap. Then the generated voltage might be electrically connected to the fabricated silicone microcapsules encapsulating the ionic liquid electrolyte in the same air gap to storage the minute electric power.

The present work is based on our previous findings^22–25^. When the ArF excimer laser irradiated silicone rubber surface, main chain of Si–O–Si bonds of silicone rubber could be photodissociated into the low molecules, resulting in the microswelling of the laser-irradiated area as follows:

$$\left( {{\text{SiO}}\left( {{\text{CH}}_{{3}} } \right)_{{2}} } \right)_{{\text{n}}} + {\text{ h}}\upnu \,\left( {\text{193 nm}} \right) \to \left( {{\text{SiO}}\left( {{\text{CH}}_{{3}} } \right)_{{2}} } \right)_{{{\text{n}} - {\text{m}}}} + \, \left( {{\text{SiO}}\left( {{\text{CH}}_{{3}} } \right)_{{2}} } \right)_{{\text{m}}}$$

To fabricate the periodic microswellings, silica glass microspheres with a diameter of 2.5 μm, which covered the entire surface of silicone rubber during laser irradiation, were used. Each the microswelled silicone rubber underneath the aligned silica glass microspheres showed a truncated cone-shape in micron size and was aligned at the regular intervals of approximately 2.5 μm. In this paper, it is found that the lower molecular weight silicones which are generated by the photodissociation are ejected from the periodic microswellings during laser irradiation along a curvature of each the microsphere to enclose the microspheres. As a result, the hollow spherical silicone microcapsule arrays after chemical etching of the enclosed microspheres can be fabricated on the periodic microswelling structures of silicone rubber, as microcontainers. Silicones are one of the useful shell wall materials because of its various good properties^26–28^.

Thus, the originality of this paper can be expressed as follows: novel fabrication process of hollow spherical silicone microcapsule arrays on silicone rubber; encapsulation of ionic liquid as electrolytes in the hollow spherical silicone microcapsules; a near-superhydrophobic property that showed on the silicone microcapsule arrays encapsulating ionic liquid; confinement of the silicone microcapsule arrays encapsulating ionic liquid in air gap of electrically insulated region under NaCl aqueous solution; simultaneous voltage generation of 0.5–0.9 V in the same air gap to supply the minute electric power to the silicone microcapsule arrays encapsulating ionic liquid. What this paper brings new compared to what already exists is that we can provide a device that combines micro-power storage and energy harvesting that can be used in seawater.

Micro-energy storage, which is convenient for combination with energy harvesting, is known to be realized by microencapsulation with various shell materials, but its application is limited to land. In this paper, we succeed in fabricating the silicone microcapsule arrays encapsulating ionic liquid electrolytes that can store minute electric power in NaCl aqueous solution as well as a minute electric power generation method. This means that each the silicone microcapsule encapsulating ionic liquid as electrolytes enables to function as an electric double layer capacitor for micro-power storage under seawater. Therefore, the fabricated samples enable to expand the range of use of Internet of Things (IoT) devices to the ocean; it can contribute to biologging in seawater for sustainable fishing and visible light communication that realizes movie transmission in seawater.

## Experimental procedure

**Figure 1:**
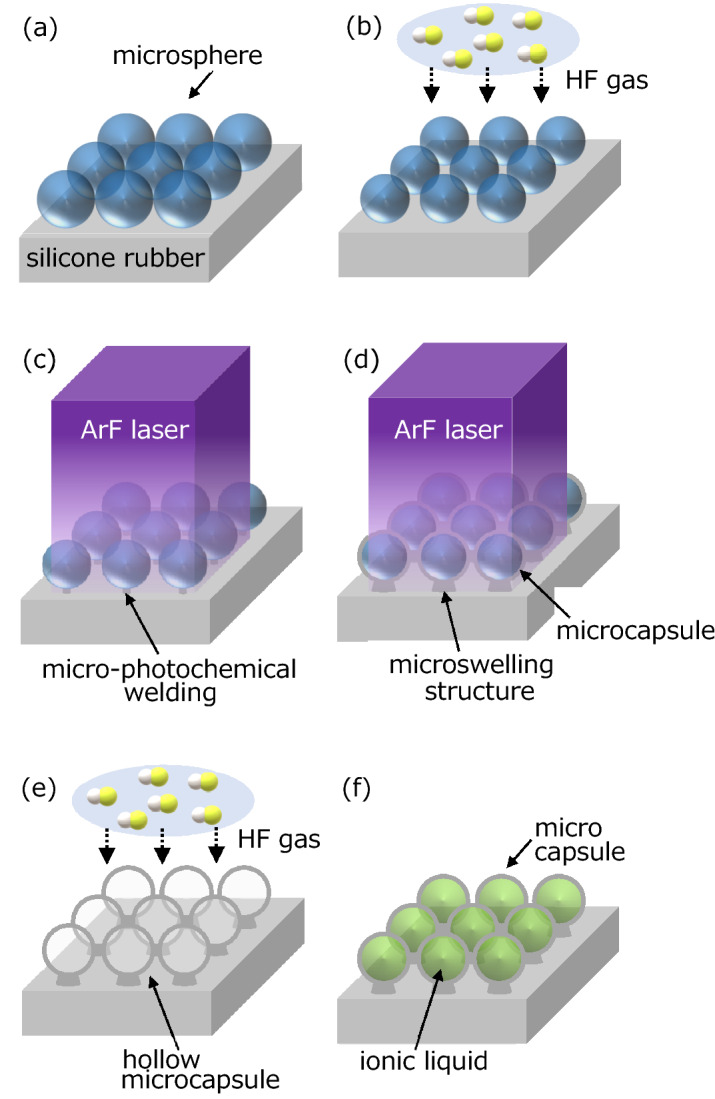
Schematic drawing of the experimental procedure: (**a**) alignment of silica glass microspheres, (**b**) pre-chemical etching of silica glass microspheres to reduce the diameter from 2.5 to 2.0 to 2.3 μm by exposure to HF gas, (**c**) pre-laser irradiation to photochemically weld silica glass microspheres to silicone rubber, (**d**) laser irradiation to fabricate microcapsules and microswelling structures on silicone rubber, (**e**) chemical etching of enclosed silica glass microspheres by exposure to HF gas, and (**f**) encapsulation of ionic liquid in the fabricated hollow silicone microcapsules under vacuum.

Figure 1 shows the schematic drawing of the experimental procedure. An appropriate amount of silica glass microspheres with a diameter of approximately 2.5 µm (Nippon Shokubai KE-P250) was taken and placed on a 2-mm-thick silicone rubber of approximately 12 × 12 mm^2^. Then the microspheres were leveled with a medicine wrapping paper. Thus, a single layer of the silica glass microspheres was formed on the silicone rubber (Fig. 1a). The sample was set to the top of the sealed beaker made of a fluorocarbon resin containing a hydrogen fluoride (HF) aqueous solution of 46–48% concentration, and the aligned silica glass microspheres that face to the HF aqueous solution were chemically etched to diameters of approximately 2.0–2.3 µm by exposure to the HF gas (Fig. 1b).

The sample was placed approximately 80 mm away from the outlet of the ArF excimer laser (Coherent COMPexPro110). The laser beam path was filled with nitrogen gas at the flow rate of 5 L/min to avoid strong optical absorption of oxygen molecules in air. To prevent the arrangement of the aligned microspheres from being disturbed during laser irradiation, the ArF excimer laser pre-irradiated the sample surface at the single pulse fluence of approximately 30 mJ/cm^2^ and at the pulse number of 100 (Fig. 1c). The pulse repetition rate was 1 Hz constant. Thus, the photodissociation of Si–O–Si bonds of silicone rubber underneath the microspheres started to occur and the microspheres could be photochemically welded to the silicone rubber^29^. Then the ArF excimer laser irradiated the pre-irradiated sample surface again at the single pulse fluence of 35–40 mJ/cm^2^ and at the pulse number of 1800 to fabricate the microcapsules and microswelling structures (Fig. 1d). All the laser irradiations were carried out at room temperature.

After the laser irradiation, the enclosed silica glass microspheres in the fabricated silicone microcapsules were chemically etched by the exposure to the HF gas (Fig. 1e). A small amount of the ionic liquid of 1-butyl-3-methylimidazolium bis(trifluoromethanesulfonyl)imide (Kanto chemical) was dripped on the fabricated hollow silicone microcapsules, then the sample was set in a vacuum. As a result, the ionic liquid could penetrate to the hollow silicone microcapsules, and the microcapsules could hold the ionic liquid stably not only in the vacuum but also in the atmosphere (Fig. 1f). The shape of the fabricated hollow microcapsule arrays was observed by the scanning electron microscope (SEM, Phenomworld, Pro). The chemical bonding states of the fabricated microcapsule arrays before and after the chemical etching of the enclosed microspheres were analyzed by the X-ray photoelectron spectroscopy (XPS, Shimadzu, KRATOS ULTRA2). The incorporation of the ionic liquid into the fabricated hollow microcapsule arrays was confirmed by the SEM and Raman spectroscopy (Jasco, NRS-5100).

## Results and discussion

**Figure 2:**
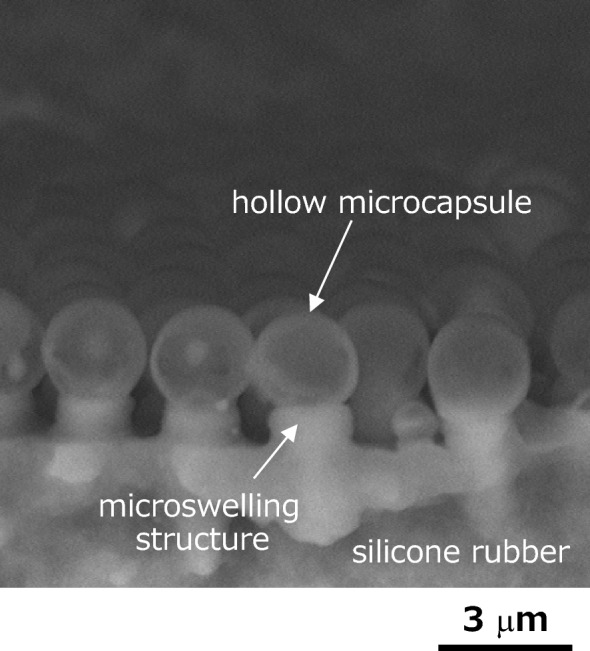
Cross-sectional SEM image of the fabricated hollow silicone microcapsules on the microswelling structures of silicone rubber.

Figure 2 shows the cross-sectional SEM image of the fabricated microcapsules and microswelling structures on silicone rubber after the exposure to HF gas. The shape of the microcapsules was almost spherical, and the diameter seems to become slightly larger, compared to the diameter of approximately 2.0–2.3 μm of the chemically etched silica glass microspheres. Though a precise shell wall thickness of the microcapsules is difficult to know accurately because the diameter of the etched microspheres is not uniform, the thickness of the fabricated microcapsules might be judged to be approximately 30 nm on average from the SEM images before and after the second laser irradiation. Underneath the microcapsules, the microswelling structure of height of approximately 1 μm was also observed and was almost uniform. In addition, the microcapsules on the microswelling structures were at the regular intervals of approximately 2.5 μm, which corresponds to the original diameter of the silica glass microspheres. When the pre-chemical etching of silica glass microspheres was not carried out, only the hemispherical silicone microcups in contact with each other could be formed^25^. This means that the lower molecular weight silicones are ejected from the microswelling structures, and it is considered that the ejected silicones could not reach the upper half of each microsphere due to the lack of gaps between microspheres due to the formation of the microcups. The fabricated microcapsules existed stably on the microswelling structures in the atmosphere even under daily vibrations. Thus, the spherical microcapsule arrays of the uniform shape, size, and height could be photochemically fabricated on the microswelling structures of silicone rubber by the 193 nm ArF excimer laser.

**Figure 3:**
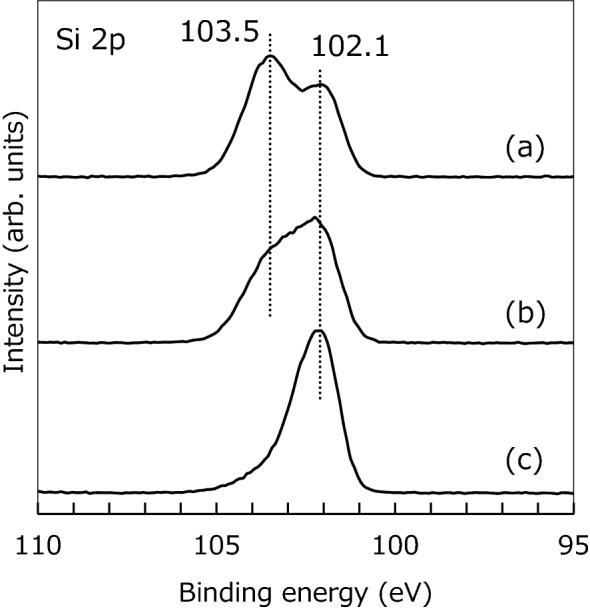
XPS spectra of the (**a**) silica glass microspheres, (**b**) fabricated silicone microcapsules enclosing silica glass microspheres, and (**c**) fabricated hollow silicone microcapsules on silicone rubbers.

To analyze the chemical bonding states of the fabricated microcapsules on silicone rubber, the XPS was conducted, as shown in Fig. 3. The analyzed area of the samples was approximately 8.5 × 10^–2^ mm^2^. When we measured the spectrum from the silica glass microspheres covering the silicone rubber surface, the peaks of Si 2p signals at 102.1 and 103. 5 eV were detected (Fig. 3a). These correspond to the Si 2p peaks originated from a silicone and a silica (SiO_2_), respectively. The reason for detecting the Si 2p peak at 102.1 eV is that the silicone rubber surface is not completely covered by the silica glass microspheres in microscopic. On the other hand, when the microcapsules enclosing the microspheres were fabricated on silicone rubber after the ArF excimer laser irradiation before the chemical etching by exposure to HF gas, both the Si 2p peaks were measured (Fig. 3b). However, the peak intensity at 103.5 eV became remarkably lower than that at 102.1 eV. This means that each the silica glass microsphere could be enclosed by the silicone molecules of lower molecular weight which were ejected from the microswelling structures by repeated the ArF excimer laser pulses. After the chemical etching by exposure to HF gas, the Si 2p peak at 103.5 eV almost disappeared; only the Si 2p peak at 102.1 eV was measured (Fig. 3c). Thus, the enclosed silica glass microspheres could be chemically etched even though there were no defined holes in the fabricated microcapsules. This might be due to a porous structure of the fabricated microcapsules. In fact, when we used a 1 wt% HF aqueous solution, the enclosed microspheres were not removed at all due to the water repelling properties of the surface. Therefore, it was found that the fabricated microcapsules were composed of silicones and could be a hollow structure applicable to a shell wall.

**Figure 4:**
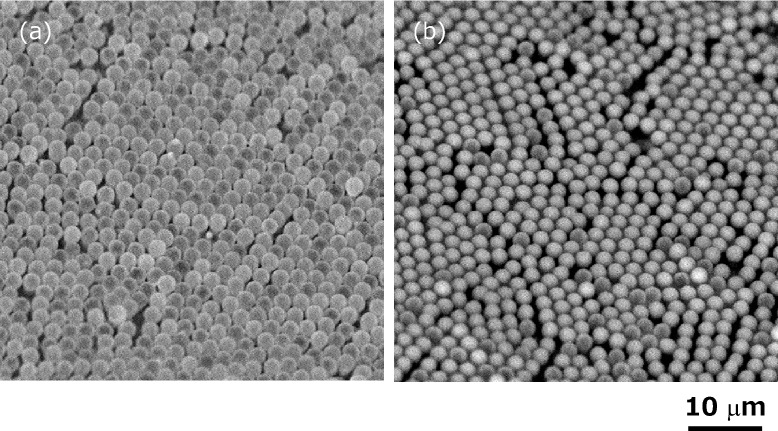
SEM images of the fabricated hollow silicone microcapsules (**a**) before and (**b**) after dripping of ionic liquid. The shade of the images on the microcapsules, the status of reflected electrons from the microcapsules, was clearly changed.

Figure 4 shows the SEM images of the fabricated hollow silicone microcapsules before and after the dripping of the ionic liquid. To achieve the microencapsulation, as a pre-treatment, the fabricated hollow silicone microcapsules were immersed in methanol (99.8% purity) as a priming solution for 5 s. Then the sample was incompletely dried at room temperature. A small amount of the ionic liquid was dripped on the half-dried sample, and the sample was set in a vacuum chamber for 30 min. Before observing the samples, in addition, the remained ionic liquid on the samples was removed by air blow. As shown in Fig. 4a,b, when the samples were observed by the SEM, shade of the images on the microcapsules, status of reflected electrons from the microcapsules, was clearly changed. The difference may have been caused by the presence or absence of the ionic liquid in the fabricated hollow silicone microcapsules. In addition, no clear change in the diameter of the microcapsules was observed before and after the dripping of the ionic liquid.

**Figure 5:**
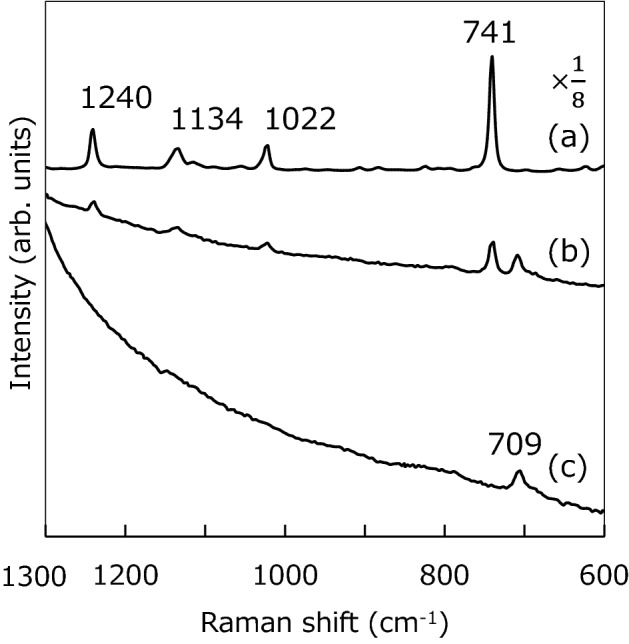
Raman spectra of the (**a**) bare ionic liquid of 1-butyl-3-methylimidazolium bis(trifluoromethanesulfonyl)imide, (**b**) fabricated silicone microcapsules encapsulating the ionic liquid, and (**c**) fabricated hollow silicone microcapsules. In case (**b**), a focal point of the 532 nm laser was needed to set to an almost center of the microcapsule.

Figure 5 shows the Raman spectra measured from the fabricated hollow silicone microcapsules before and after the dripping of the ionic liquid. In the measurements, a 532 nm wavelength of a laser was used for excitation. A range of the measured Raman shift was set to 600–1300 cm^−1^. For reference, a Raman spectrum of the bare ionic liquid was measured; the peaks of Raman shift at 741, 1022, 1134, and 1240 cm^−1^ were detected (Fig. 5a). In the case of the hollow silicone microcapsules, the peak was at 709 cm^−1^ (Fig. 5c). This peak was agreed with one of the peaks at 685, 709, 787, 860 and 1261 cm^−1^ of a bare silicone rubber. This might be because the peak at 709 cm^−1^ was extremely strong among the five peaks. Thus, in the case of microcapsules, it was difficult to measure clearly except for the peak at 709 cm^−1^. When the ionic liquid was dripped on the fabricated hollow silicone microcapsules in a vacuum, the peaks at 741, 1022, 1134, and 1240 cm^−1^ for the ionic liquid were clearly measured, together with a peak of silicone at 709 cm^−1^ (Fig. 5b). In this case, a focal point of the 532 nm laser was needed to set to an almost center of the microcapsule. Therefore, each the fabricated hollow silicone microcapsule could be successfully filled with the ionic liquid. In addition, the encapsulated ionic liquid was stable in the atmosphere at room temperature without leaking out of the microcapsules.

Based on the above results, model development for the fabrication process of silicone microcapsule arrays encapsulating ionic liquid might be possible. The ArF excimer laser of 193 nm wavelength is essential for the photodissociation of Si–O–Si bonds of silicone rubber, which occurs effectively underneath the silica glass microspheres that work as microlenses. The photodissociation induces the fabrication of the microswelling structures of silicone rubber, and subsequent ejection of the lower molecular weight silicones take place. Preparation of appropriated gaps between microspheres is needed to be enclosed the microspheres by the ejected lower molecular weight silicones to fabricate the silicone microcapsule arrays on the microswelling structures. The microspheres also function as a template for fabricating the spherical microcapsules. As the fabricated silicone microcapsules are a porous structure, the enclosed microspheres can be chemically etched by the HF gas. Moreover, the ionic liquid can be also penetrated in the fabricated hollow silicone microcapsules in a vacuum, together with a small amount of methanol as a priming solution.

For practical use, however, it is necessary to improve the fabrication process; flatness of silicone rubber surface, automation of microsphere alignment (packing density of microspheres) and etching processes (etching uniformity), and beam homogenization of ArF excimer laser. Namely, a stage is required to evaluate the fabrication process designed to ensure it can reproduce consistent and reliable levels of quality for practical use. It involves collecting and evaluating data on all aspects and stages of the fabrication process as described above. By validating the above data, it is considered that a uniform silicone microcapsule arrays with high reproducibility can be fabricated over a wide area on silicone rubber.

The meaning that the fabricated silicone microcapsules of the uniform shape, size and height are fixed to silicone rubber is to be showed a superhydrophobic or near-superhydrophobic property on the surface as mentioned below. As a result, when the samples are put under an aqueous solution, it is expected to be formed an air gap on the superhydrophobic or near-superhydrophobic silicone rubber^23,30^. Thus, we came up with the idea that the fabricated silicone microcapsules encapsulating a conductive ionic liquid enable to be confined in the air gap which gives an electrically insulated region in an aqueous solution. Considering the ionic liquid as electrolytes, the fabricated silicone microcapsules encapsulating the ionic liquid suggest the possibility of realizing micro-electric double layer capacitors, which means that the expected device might be used for micro-power storage under aqueous solution.

**Figure 6:**
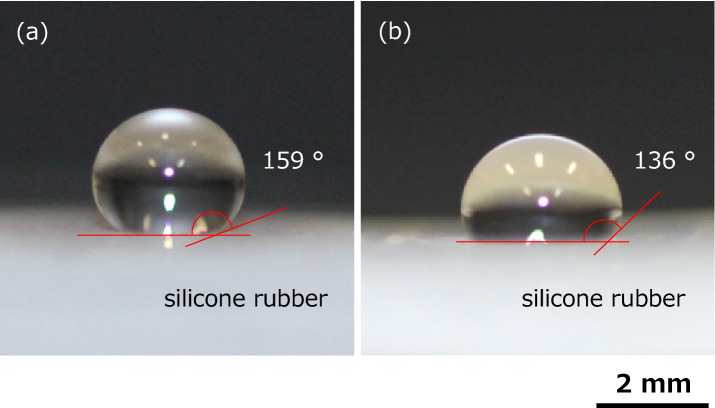
Cross-sectional photographs of the water droplet on the (**a**) fabricated hollow silicone microcapsules and (**b**) fabricated silicone microcapsules encapsulating the ionic liquid, for the measurement of contact angle of water.

**Figure 7:**
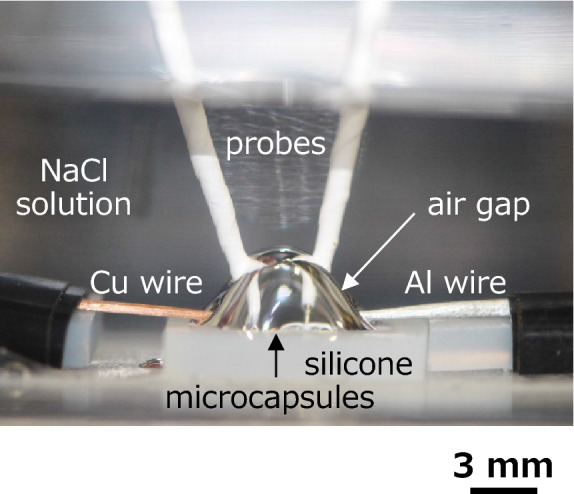
Photograph of the inflated air gap of electrically insulated region formed on the near-superhydrophobic silicone microcapsule arrays under a 3 wt% NaCl aqueous solution, together with the generation of electric voltage between Al and Cu wires in the same air gap. A couple of probes were inserted into the inflated air gap and touched to each metal wire to detect the electric voltage generated.

Toward demonstrating the expected device for micro-power storage under aqueous solution, we measured the contact angle of water on the fabricated silicone microcapsules. For comparison, the contact angle of water was approximately 90 degrees on a bare silicone rubber. On the other hand, the contact angle was estimated to be approximately 159 and 136 degrees on the fabricated silicone microcapsules before and after the encapsulating ionic liquid, respectively (Fig. 6a,b). These indicate a superhydrophobic or near-superhydrophobic property. The reason why the contact angle decreases after encapsulating ionic liquid is that it is difficult to remove the ionic liquid remaining in the gaps of the microcapsules during dripping. When the near-superhydrophobic sample was put slowly under a 3 wt% NaCl aqueous solution, a uniform air gap was formed on the sample surface. Also, the air gap can be inflated to expand the electrically insulated region by injection of air with a sringe^21,23,30^. Figure 7 shows the photograph of the inflated air gap formed on the silicone microcapsules in the NaCl aqueous solution. The shape of this inflated air gap was slightly changed by inserting probes and aluminum (Al) and copper(Cu) wires, compared to immediately after inflated air gap formation^21^. The fabricated silicone microcapsules could be confined in the inflated air gap. Moreover, as shown in Fig. 7, when the Al and Cu wires were provided across the air gap and NaCl aqueous solution on the near-superhydrophobic sample, electric voltages of 0.5–0.9 V between two wires could be obtained by insertion of a pair of probes. The generation of electric voltages is based on the difference in the electrochemical potentials of two metal wires, and a standard electrode potential of Al is as follows:

$${\text{Al}}^{{3+}} + {\text{3e}}^{ - } \to {\text{Al }}\left( {{\text{E}}^{0} = \, - {1}.{\text{676 V}}} \right)$$

On the other hand, the following reaction is thought to occur at the Cu electrode:

$${\text{2H}}_{{2}} {\text{O}} + {\text{2e}}^{ - } \to {\text{H}}_{{2}} + {\text{2OH}}^{ - }\, \left( {{\text{E}}^{0} = \, - 0.{\text{828 V}}} \right)$$

Thus, an electric voltage of 0.848 V is ideally expected, which is in the obtained electric voltages of 0.5–0.9 V. Therefore, it is suggested that the obtained electric voltage can be electrically connected to the fabricated silicone microcapsules encapsulating ionic liquid. To realize the micro-electric double layer capacitors, it is necessary to form electrodes inside the silicone microcapsules and to make electrical connections between the silicone microcapsules. After overcoming this challenge, the present device for micro-energy storage opens up the possibility to realize the IoT devices that work under seawater. Moreover, the academic question that forms the core of this research is that whether it is possible to find the solution that were previously considered difficult to achieve, by designing the space that exists at the interface between contradictory substances, such as a superhydrophobic surface and water. This paper can provide the solution that it is the manifestation of electrical functions on the surface of materials in seawater.

## Conclusions

The hollow spherical silicone microcapsule arrays of the uniform shape, size, and height were successfully fabricated on the silicone rubber by the 193 nm ArF excimer laser. The laser-irradiated silicone rubber underneath the aligned silica glass microspheres was photochemically and periodically swelled by the photodissociation of Si–O–Si bonds of the silicone rubber. Accompanied by the photochemical microswellings, the lower molecular weight silicones which were generated by the photodissociation were ejected from the microswelling structures along the curvature of each the microsphere to enclose the microspheres. After the chemical etching of the enclosed silica glass microspheres by the exposure to the HF gas, the fabricated silicone microcapsules became hollow, which was confirmed by the XPS. Moreover, each the hollow silicone microcapsule could entrap the ionic liquid in the vacuum. The incorporation of the ionic liquid into the hollow silicone microcapsules was observed by the SEM and Raman spectroscopy. As the fabricated silicone microcapsule arrays before and after the encapsulating the ionic liquid were fixed to the silicone rubber and showed the superhydrophobic or near-superhydrophobic property, the silicone microcapsules encapsulating the ionic liquid as electrolytes could be confined in the inflated air gap of the electrically insulated region formed on the near-superhydrophobic samples in the NaCl aqueous solution. Also, based on the difference in electrochemical potentials, the combination of our previous method allowed the electric voltages of 0.5–0.9 V to be generated simultaneously in the air gap. Therefore, the generated electric voltage might be electrically connected to the silicone microcapsule arrays encapsulating the ionic liquid electrolyte in the same air gap under the NaCl aqueous solution. This means that each the silicone microcapsule encapsulating ionic liquid as electrolytes enables to function as the electric double layer capacitor that stores minute power in seawater and at the same time can generate and supply minute power using the seawater environment. In addition, by numerical approach, it may be possible to find the optimal microsphere size before the pre-chemical etching that can improve the near-superhydrophobic property of silicone microcapsules encapsulating ionic liquid to superhydrophobic property. The present results enable to expand the range of use of IoT devices to the ocean and will also lead to the realization of microdevices that perform visible light communication in seawater.

## Data Availability

The data that support the findings of this research are available from the corresponding author upon reasonable request.
