# Publisher Correction: Near-superhydrophobic silicone microcapsule arrays encapsulating ionic liquid electrolytes for micro-power storage assuming use in seawater

**DOI:** 10.1038/s41598-022-25424-7

**Published:** 2022-12-09

**Authors:** Kaede Iwasaki, Tsuyoshi Yoshida, Masayuki Okoshi

**Affiliations:** Department of Electrical and Electronic Engineering, National Defense Academy, 1-10-20 Hashirimizu, Yokosuka, Kanagawa 239-8686 Japan

Correction to: *Scientific Reports* 10.1038/s41598-022-22891-w, published online 29 October 2022

The original version of this Article contained errors in the legends of Figure 4, 5 and 6. The legends were inadvertently switched.

The legend of Figure 4:

“Cross-sectional photographs of the water droplet on the (a) fabricated hollow silicone microcapsules and (b) fabricated silicone microcapsules encapsulating the ionic liquid, for the measurement of contact angle of water.”

now reads:

“SEM images of the fabricated hollow silicone microcapsules (a) before and (b) after dripping of ionic liquid. The shade of the images on the microcapsules, the status of reflected electrons from the microcapsules, was clearly changed.”

The legend of Figure 5:

“SEM images of the fabricated hollow silicone microcapsules (a) before and (b) after dripping of ionic liquid. The shade of the images on the microcapsules, the status of reflected electrons from the microcapsules, was clearly changed.”

now reads:

“Raman spectra of the (a) bare ionic liquid of 1-butyl-3-methylimidazolium bis (trifluoromethanesulfonyl)imide, (b) fabricated silicone microcapsules encapsulating the ionic liquid, and (c) fabricated hollow silicone microcapsules. In case (b), a focal point of the 532 nm laser was needed to set to an almost center of the microcapsule.”

The legend of Figure 6:

“Raman spectra of the (a) bare ionic liquid of 1-butyl-3-methylimidazolium bis (trifluoromethanesulfonyl)imide, (b) fabricated silicone microcapsules encapsulating the ionic liquid, and (c) fabricated hollow silicone microcapsules. In case (b), a focal point of the 532 nm laser was needed to set to an almost center of the microcapsule.”

now reads:

“Cross-sectional photographs of the water droplet on the (a) fabricated hollow silicone microcapsules and (b) fabricated silicone microcapsules encapsulating the ionic liquid, for the measurement of contact angle of water.”

The original Article has been corrected.

